# Regulation of Th17 Cytokine-Induced Osteoclastogenesis via SKI306X in Rheumatoid Arthritis

**DOI:** 10.3390/jcm8071012

**Published:** 2019-07-10

**Authors:** Hae-Rim Kim, Kyoung-Woon Kim, Bo-Mi Kim, Ji-Yeon Won, Hong-Ki Min, Kyung-Ann Lee, Tae-Young Kim, Sang-Heon Lee

**Affiliations:** 1 Division of Rheumatology, Department of Internal Medicine, Research Institute of Medical Science, School of Medicine, Konkuk University, Seoul 05030, Korea; 2 Conversant Research Consortium in Immunologic Disease, Seoul St. Mary’s Hospital, The Catholic University of Korea, Seoul 06591, Korea; 3 Division of Rheumatology, Department of Internal Medicine, Soonchunhyang University Hospital, Seoul 04401, Korea; 4 Department of Orthopedic Surgery, School of Medicine, Konkuk University, Seoul 05030, Korea

**Keywords:** rheumatoid arthritis, synovial fibroblasts, cytokine, osteoclast, herbal medicine

## Abstract

This study aimed to investigate the regulatory effect of SKI306X, a mixed extract of three herbs, in T helper (Th)17 cytokine-induced inflammation and joint destruction in rheumatoid arthritis (RA). Synovial fibroblasts were isolated from RA patients and cultured with Th17 cytokines including interleukin (IL)-17, IL-21, and IL-22 and SKI306X, and tumor necrosis factor (TNF)-α, IL-1β, and receptor activator of nuclear factor kappa-Β ligand (RANKL) expression and production were investigated using real-time PCR and ELISA of culture media. After peripheral blood (PB) cluster of differentiation (CD)14^+^ monocytes were cultured in media supplemented with Th17 cytokines and SKI306X, tartrate-resistant acid phosphatase positive (TRAP^+^) multinucleated giant cells (mature osteoclasts) were enumerated and gene expression associated with osteoclast maturation was assessed via real-time PCR analysis. After PB monocytes were co-cultured with IL-17-stimulated RA synovial fibroblasts in the presence of SKI306X, osteoclast differentiation was assessed. When RA synovial fibroblasts were cultured with IL-17, IL-21, and IL-22, TNF-α, IL-1β, and RANKL expression and production were increased; however, SKI306X reduced cytokine expression and production. When PB monocytes were cultured in media supplemented with Th17 cytokines, osteoclast differentiation was stimulated; however, SKI306X decreased osteoclast differentiation and osteoclast maker expression. When PB monocytes were co-cultured with IL-17-stimulated RA synovial fibroblasts, osteoclast differentiation was increased; however, SKI306X decreased osteoclast differentiation and osteoclast maker expression. SKI306X reduced Th17 cytokine-induced TNF-α, IL-1β, and RANKL expression and osteoclast differentiation, providing novel insights into adjuvant therapy for regulating inflammation and joint destruction in RA.

## 1. Introduction

A few decades ago, Korean patients with rheumatoid arthritis (RA) received herbal medication and acupuncture before they visited rheumatologic clinics. They believed the herbal therapies could cure RA without any adverse effects. By the time these patients visited rheumatologists, they already had joint destruction and disability because of delayed treatment with disease-modifying antirheumatic drugs (DMARDs). RA is currently considered an autoimmune inflammatory disease requiring treatment with DMARDs, which has led to an increase in rheumatologic consultations. However, DMARDs do not always have therapeutic effects in RA, they can display adverse effects and are highly expensive [1]. Hence, some patients prefer complementary and alternative medication, primarily including herbal medication [1,2]. According to the Korean RA registry (KORONA), 10.5% of patients received complementary and alternative medicines de novo and among them, 17% received herbal medication and 55% received acupuncture [3].

SKI306X, a mixed extract of three herbs, is a purified extract prepared from a mixture of three Oriental herbal plants, *Clematis mandshurica*, *Trichosanthes kirilowii*, and *Prunella vulgaris* [4]. According to the Donguibogam, i.e., ‘Principles and Practice of Eastern Medicine’ written in 1610, *Clematis mandshurica* effectively reduces lower back and knee pain, *Trichosanthes kirilowii* reduces febrile sensations and dry mouth, and *Prunella vulgaris* effectively reduces lymphadenitis, abscess, and ulcers. The anti-inflammatory effects of SKI306X have been reported previously.

SKI306X inhibits tumor necrosis factor (TNF)-α, leukotriene B4, and nitric oxide (NO) production in macrophages and cyclooxygenase-2 expression [5]. It inhibits TNF-α, prostaglandin E2, and interleukin (IL)-1β production by stimulated peripheral blood (PB) mononuclear cells [6] and is commonly used to treat osteoarthritis owing to its chondroprotective effects. SKI306X inhibits the IL-1β-induced production of proteoglycans, NO, matrix metalloproteinases (MMPs) and degradation of glycosaminoglycan by cartilage [4,6]. A preclinical animal study reported that SKI306X protects against osteoarthritis. Collagenase injection at the knee joint of rabbits led to osteoarthritis-like degeneration of articular cartilage and development of synovial tissues; however, SKI306X reduces osteoarthritis-like histological changes [4]. In chondrocytes cultured in media supplemented with IL-1α, the cumulative activity of MMP-3 and MMP-13 increased; however, SKI306X significantly reduced their activities and inhibited the activation of the proenzyme MMP-3 to the active MMP-3 [7]. In clinical trials of knee osteoarthritis, SKI306X treatment resulted in greater changes in pain and function than the placebo [8,9]. The change in cartilage volume and thickness of the lateral tibia were reportedly greater after treatment with SKI306X than the placebo [8].

Two studies have reported that SKI306X has similar effects in controlling for pain and disease activity and has greater cardiovascular safety than celecoxib in RA. When SKI306X was compared with celecoxib among RA patients, SKI306X was not inferior to celecoxib with regards to pain score (visual analog scale), American College of Rheumatology (ACR) 20 response rate, frequency of rescue medication, and drug-related adverse effects [10]. Unlike celecoxib, SKI306X does not have a higher risk of cardiovascular events in patients with RA and osteoarthritis [11]. However, the limitation of this clinical study was that celecoxib is not a DMARD and it cannot prevent joint destruction. Thus far, no study has investigated the anti-inflammatory and joint protective effects of SKI306X in RA. Therefore, this study aimed to investigate the regulatory effect of SKI306X in T helper (Th)17 cytokine-induced inflammation and bone destruction in RA.

## 2. Methods

### 2.1. Patients

Informed consent was obtained from all patients, and the experimental protocol was approved by the Institutional Review Board for Human Research, Konkuk University Hospital (KUH1010186, approved on January 22nd 2010). Synovial tissues were isolated from eight RA patients (mean age 63.4 ± 4.6 years, range 38–76 years) undergoing total knee and hip replacement surgery.

### 2.2. Isolation of Synovial Fibroblasts

Synovial fibroblasts were isolated by enzymatic digestion of synovial tissues, as described previously [12,13]. Synovial fibroblast cell lines were prepared from synovectomized tissue of RA patients undergoing joint replacement surgery. To set up cell lines, synovial tissues were minced into 2–3 mm pieces and treated for 4 h with 4 mg/mL type 1 collagenase (Worthington Biochemicals, Freehold, NJ, USA) in Dulbecco’s modified Eagle’s medium (DMEM) at 37 °C in 5% CO_2_. Dissociated cells were centrifuged at 500× *g* and were resuspended in DMEM supplemented with 10% fetal calf serum (FCS), 2 mM l-glutamine, 100 U/mL penicillin, and 100 μg/mL streptomycin. Suspended cells were plated in 75 cm^2^ culture flasks and cultured at 37 °C in 5% CO_2_. Medium was replaced every 3 days, and once the primary culture reached confluence, cells were split weekly. Cells at passages 5 to 8 contained a homogeneous population of synovial fibroblasts (<2.5% CD14 positive, <1% CD3 positive, and <1% CD19 positive in flow cytometric analysis).

### 2.3. Reagents

Recombinant human IL-17 (20 ng/mL), IL-21 (20 ng/mL), IL-22 (20 ng/mL), and macrophage colony-stimulating factor (M-CSF) (25 ng/mL) were purchased from R&D Systems (Minneapolis, MN, USA). SKI306X (at 0 μg/mL, 1 μg/mL, 5 μg/mL and 10 μg/mL) were generously provided by the Life Science R&D Center of SK Chemicals (Seongnam, Korea). Anti-TNF-α, anti-IL-1β and anti-RANKL antibodies were purchased from R&D Systems.

### 2.4. Real-Time PCR

Synovial fibroblasts were stimulated with IL-17, IL-21, or IL-22. Synovial fibroblasts were incubated in the presence or absence of SKI306X for 3 h before the addition of IL-17, IL-21, or IL-22. After incubation for 72 h, mRNA was extracted using RNAzol B (Biotex Laboratories, Houston, TX, USA) according to the manufacturer’s instructions.

### 2.5. Enzyme-Linked Immunosorbent Assay (ELISA)

TNF-α, IL-1β, IL-8 and soluble receptor activator of nuclear factor kappa-Β ligand (sRANKL) levels in the culture supernatants from RA synovial fibroblasts were measured using a sandwich ELISA according to R&D System’s instructions.

### 2.6. Osteoclast Formation

PB monocytes were prepared from heparinized blood by Ficoll–Hypaque (GE Healthcare, Pittsburgh, PA, USA) density gradient centrifugation. Monocytes were added to the IL-17-pretreated RA synovial fibroblasts with fresh media. Monocytes were co-cultured for 3 weeks in α-minimal essential medium and 10% fetal bovine serum (FBS) in the presence of 25 ng/mL recombinant human macrophage-colony stimulating factor (rhM-CSF). On day 21, tartrate-resistant acid phosphatase (TRAP)-positive cells were identified using a leukocyte acid phosphatase kit according to Sigma-Aldrich’s protocol [14].

### 2.7. Statistical Analysis

The data are expressed as means ± standard deviation (SD). Statistical difference was assessed using Mann–Whitney *U* test for analyzing two groups or one-way analysis of variance (ANOVA) with Bonferroni’s multiple comparison post-hoc test for analyzing more than three groups. A *p* value < 0.05 was considered statistically significant.

## 3. Results

### 3.1. Regulatory Effect of SKI306X on Th17 Cytokine-Induced TNF-α Expression and Production in RA Synovial Fibroblasts

**Figure 1:**
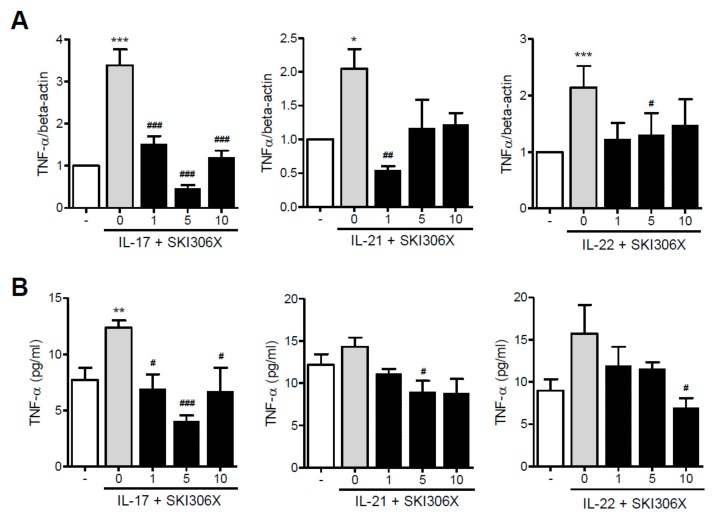
The inhibitory effect of SKI306X, a mixed extract of three herbs, in T helper (Th)17 cytokine-induced tumor necrosis factor (TNF)-α expression and production by synovial fibroblasts in rheumatoid arthritis (RA). (**A**) RA synovial fibroblasts were cultured in media supplemented with interleukin (IL)-17, IL-21, and IL-22 in the presence of various doses of SKI306X, and TNF-α was examined using real-time PCR, normalized to that of beta-actin and reported in relative expression units. (**B**) RA synovial fibroblasts were cultured with IL-17, IL-21, and IL-22 in the presence of various concentrations of SKI306X, and TNF-α production in culture media was determined using ELISA. The data represent the mean ± standard deviation (SD) values from six independent experiments. * *p* < 0.05, ** *p* < 0.01 and *** *p* < 0.001 compared with the nil condition (white bars). # *p* < 0.05, ## *p* < 0.01 and ### *p* < 0.001 compared with the Th17 cytokine stimulating condition (gray bars).

When RA synovial fibroblasts were cultured in media supplemented with IL-17, IL-21, or IL-22, TNF-α was upregulated; however, SKI306X reduced Th17 cytokine-induced TNF-α expression (Figure 1A), TNF-α production was increased upon IL-17 stimulation in culture media, and SKI306X reduced TNF-α production (Figure 1B).

### 3.2. The Regulatory Effect of SKI306X on Th17 Cytokine-Induced IL-1β Expression and Production in RA Synovial Fibroblasts

**Figure 2:**
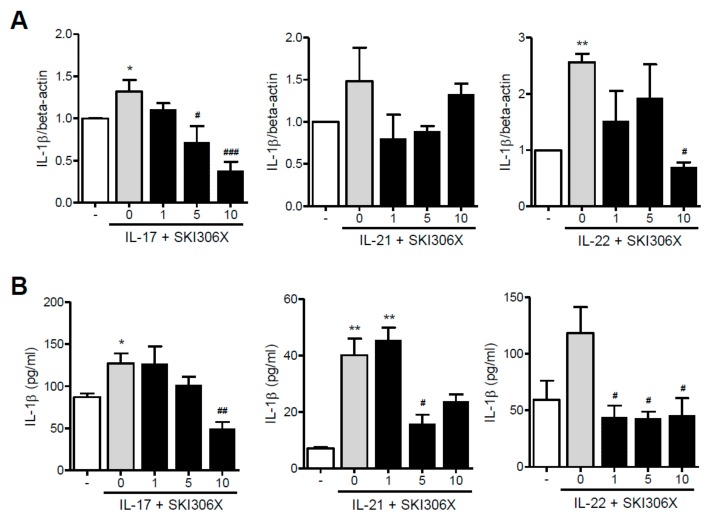
The inhibitory effect of SKI306X in Th17 cytokine-induced IL-1β expression and production in synovial fibroblasts in RA. (**A**) RA synovial fibroblasts were cultured in medium supplemented with IL-17, IL-21, and IL-22 with various concentrations of SKI306X, and IL-1β expression was examined using real-time PCR, normalized to beta-actin and reported in relative expression units. (**B**) RA synovial fibroblasts were cultured in medium supplemented with IL-17, IL-21, and IL-22 in the presence of various doses of SKI306X, and IL-1β production in the culture media was determined via ELISA. The data represent mean ± SD values from six independent experiments. * *p* < 0.05 and ** *p* < 0.01 compared with the nil condition (white bars). # *p* < 0.05, ## *p* < 0.01 and ### *p* < 0.001 compared with the Th17 cytokine stimulating condition (gray bars).

When RA synovial fibroblasts were cultured in media supplemented with IL-17 or IL-22, IL-1β was upregulated; however, SKI306X reduced Th17 cytokine-induced IL-1β expression. Stimulation with IL-21 resulted in a similar, albeit non-significant, effect (Figure 2A). Furthermore, IL-1β production was increased after IL-17 or IL-21 supplementation in culture media, and SKI306X reduced IL-1β production (Figure 2B).

However, IL-21 and IL-22 did not promote IL-8 expression and production. Although IL-17 induced IL-8 expression and production, SKI306X inhibited IL-17-induced IL-8 production in RA synovial fibroblasts but did not affect IL-17-induced IL-8 expression.

### 3.3. Regulatory Effect of SKI306X on Th17 Cytokine-Induced RANKL Expression and Production in RA Synovial Fibroblasts

**Figure 3:**
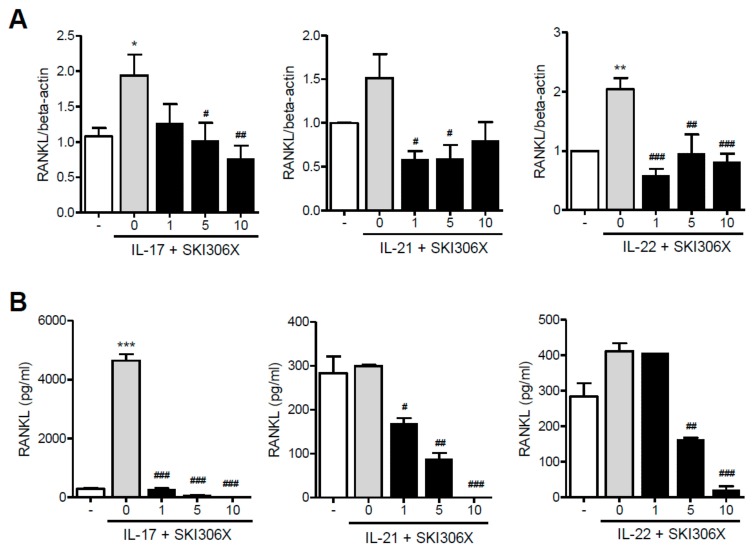
The inhibitory effect of SKI306X in Th17 cytokine-induced receptor activator of nuclear factor kappa-Β ligand (RANKL) expression and production in synovial fibroblasts in RA. (**A**) RA synovial fibroblasts were cultured in medium supplemented with IL-17, IL-21, and IL-22 with various concentrations of SKI306X, and RANKL expression was examined using real-time PCR, normalized to beta-actin and reported in relative expression units. (**B**) RA synovial fibroblasts were cultured in medium supplemented with IL-17, IL-21, and IL-22 with various concentrations of SKI306X, and RANKL production in the culture media was examined using ELISA. The data represent mean ± SD values from six independent experiments. * *p* < 0.05, ** *p* < 0.01 and *** *p* < 0.001 compared with the nil condition (white bars). # *p* < 0.05, ## *p* < 0.01 and ### *p* < 0.001 compared with the Th17 cytokine stimulating condition (gray bars).

On culturing RA synovial fibroblasts in media supplemented with IL-17 or IL-22, RANKL was upregulated; however, SKI306X reduced Th17 cytokine-induced RANKL expression (Figure 3A). Furthermore, RANKL production was increased upon IL-17 supplementation in culture media, and SKI306X reduced IL-17-induced RANKL production (Figure 3B).

### 3.4. Regulatory Effect of SKI306X on Th17 Cytokine-Induced Osteoclast Differentiation from PB Monocytes

**Figure 4:**
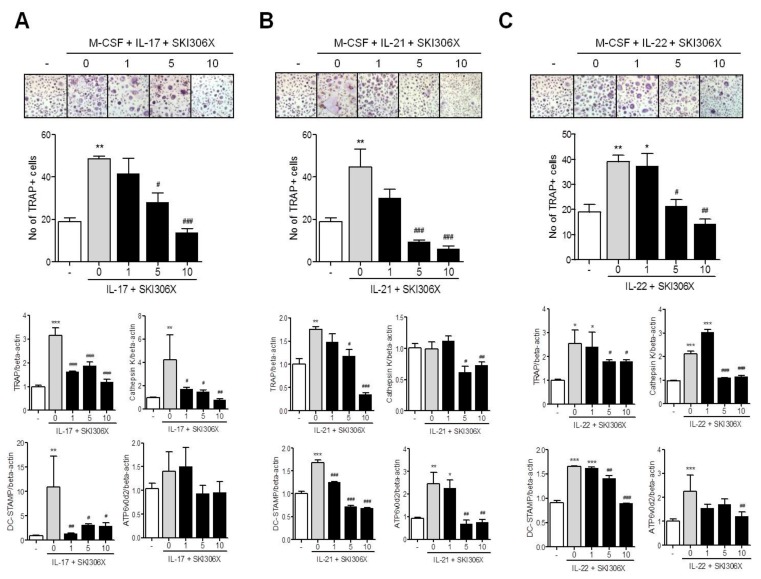
Regulatory effect of SKI306X on Th17 cytokine-induced osteoclast differentiation in peripheral blood (PB) monocytes. PB cluster of differentiation (CD)14^+^ monocytes were cultured in medium supplemented with (**A**) IL-17, (**B**) IL-21, or (**C**) IL-22 and various concentrations of SKI306X in the presence of 25 ng/mL of monocyte colony stimulating factor. After 21 days of culturing, TRAP-positive multinucleated cells were enumerated. The figures represent one of three independent experiments and the bars represent mean ± SD values. The expression of osteoclast markers including tartrate-resistant acid phosphatase (TRAP), cathepsin K, dendritic cell specific transmembrane protein (DC-STAMP), and ATP6v0d2 was quantified using real-time PCR, normalized to beta-actin and reported in relative expression units. * *p* < 0.05, ** *p* < 0.01 and *** *p* < 0.001 compared with the nil condition (white bars). # *p* < 0.05, ## *p* < 0.01 and ### *p* < 0.001 compared with the Th17 cytokine stimulating condition (gray bars).

When CD14^+^ monocytes isolated from the PB of healthy donors were cultured in media supplemented with IL-17 and monocyte colony stimulating factor (M-CSF), TRAP-positive multinucleated osteoclasts were differentiated, and SKI306X reduced osteoclast differentiation in a dose-dependent manner. Osteoclast markers including TRAP, cathepsin K, and dendritic cell specific transmembrane protein (DC-STAMP) were upregulated upon IL-17 stimulation, and SKI306X downregulated these factors (Figure 4A). Monocyte stimulation with IL-21 or IL-22 yielded a similar pattern as IL-17 during osteoclast differentiation. On culturing CD14^+^ monocytes in media supplemented with IL-21 or IL-22 and M-CSF, TRAP-positive multinucleated osteoclasts differentiated, and SKI306X reduced osteoclast differentiation in a dose-dependent manner. Osteoclast markers including TRAP, cathepsin K, DC-STAMP, and ATP6v0d2 were also upregulated upon IL-21 and IL-22 stimulation, and SKI306X reduced their expression (Figure 4B,C).

### 3.5. Regulatory Effect of SKI306X on Osteoclast Differentiation from PB Monocytes Co-Cultured with IL-17-Stimulated RA Synovial Fibroblasts

**Figure 5:**
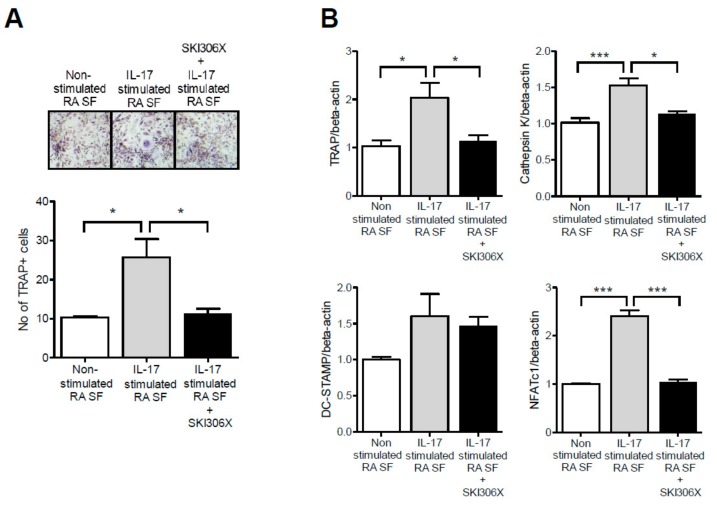
The regulatory effect of SKI306X on osteoclast differentiation in co-cultures of PB monocytes and IL-17-stimulated synovial fibroblasts (SF) in RA. (**A**) After PB monocytes were co-cultured with IL-17 stimulated RA synovial fibroblasts in the presence of monocyte colony stimulating factor and SKI306X for 21 days, the TRAP-positive multinucleated cells were enumerated. The figures represent one of three independent experiments and the bars represent mean ± SD values. (**B**) The expression of osteoclast markers including TRAP, cathepsin K, DC-STAMP, and nuclear factor of activated T-cells, cytoplasmic 1 (NF-ATc1) was quantified using real-time PCR, normalized to beta-actin and reported in relative expression units. * *p* < 0.05 and *** *p* < 0.001.

CD14^+^ monocytes isolated from PB were co-cultured with IL-17-prestimulated RA synovial fibroblasts in media supplemented with M-CSF, and TRAP-positive multinucleated osteoclasts were differentiated and compared with non-stimulated RA synovial fibroblasts. SKI306X reduced osteoclast differentiation (Figure 5A). Furthermore, osteoclast markers including TRAP, cathepsin K, and nuclear factor of activated T-cells, cytoplasmic 1 (NF-ATc1) were upregulated when osteoclast precursors were co-cultured with IL-17-prestimulated RA synovial fibroblasts, and SKI306X reduced their expression (Figure 5B).

## 4. Discussion

In some Asian countries, herbal medicines are commonly used for treating arthritis. Although many patients do not receive prompt diagnosis and treatment for RA, some patients have experienced therapeutic effects using herbal medication [1,3]. SKI306X is widely used to manage osteoarthritis in Korea; however, no study has reported the efficacy of herbal medication in RA. This study aimed to investigate the anti-inflammatory and osteoprotective effects of SKI306X in Th17 cytokine-induced inflammation and osteoclast differentiation in RA.

To determine the anti-inflammatory effect of SKI306X, we examined Th17 cytokine-induced TNF-α and IL-1β expression in RA synovial fibroblasts. We selected synovial fibroblasts and Th17 cytokines in this study because they are primary targets in RA pathogenesis; however, they are very active effector cells, which can induce and aggravate inflammatory processes [15,16]. We previously reported that IL-21, IL-22, and IL-17 stimulate RA synovial fibroblasts to produce inflammatory cytokines [13,17,18]. Moreover, Th17 overproduction and the differentiation of Th17-positive cells are one of the major events in RA pathogenesis; hence, reduction of the Th17 response regulates inflammation and joint destruction in RA [19,20]. IL-17, IL-21, and IL-22 upregulated TNF-α and IL-1β in RA synovial fibroblasts and SKI306X effectively reduced their expression. A previous study reported that SKI306X reduces lipopolysaccharide (LPS)-induced TNF-α and IL-1β production in human peripheral blood mononuclear cells (PBMCs) [20]. Another study reported that SKI306X inhibits TNF-α release from LPS-stimulated human whole blood; however, it does not affect IL-1β release [5]. LPS is a nonspecific stimulant in the inflammatory processes. In this study, we used RA synovial fibroblasts, which represent target cells, and stimulated them with Th17 cytokines as specific stimulators and LPS as a non-specific cellular stimulator. Thus, we replicated pathological conditions in vitro and investigated the therapeutic effect of SKI306X. Reduction of Th17-induced TNF-α and IL-1β expression and production by SKI306X indicates that SKI306X plays a potential anti-inflammatory role in Th17-induced inflammation in RA.

Furthermore, we determined the bone protective role of SKI306X in RA. In RA, loss of cartilage and bone erosion cause joint destruction and subsequent joint disability. Bone erosion is caused by bone resorption of synovial osteoclasts, which in turn are activated by RANKL and other inflammatory cytokines. They originate from synovial tissues, subchondral bone, and circulating monocytes in inflammatory conditions [21]. We examined the dual roles of SKI306X in the reduction of RANKL production from synovial fibroblasts and in the inhibition of osteoclast differentiation. Because synovial fibroblasts are major sources of RANKL [13,22,23], SKI306X inhibited Th17 cytokine-induced RANKL expression and production in RA synovial fibroblasts. These findings suggest that SKI306X potentially ameliorates bone destruction because RANKL is a very important molecule in osteoclast activation. In recent clinical trials, denosumab, an anti-RANKL antibody, inhibited the progression of joint destruction in RA patients [24,25]. Although denosumab does not reduce inflammation, combinatorial treatment with conventional or biologic DMARDs can effectively reduce both inflammation and joint destruction.

Finally, we examined the inhibitory effect of SKI306X in osteoclast differentiation. RA treatment is primarily aimed at preventing joint destruction; hence, inhibition of osteoclastogenesis is critical for treatment. Circulating CD14^+^ monocytes are precursors of osteoclasts and they upregulate RANK on the cell surface and interact with RANKL that is primarily produced by RA synovial fibroblasts and Th17 cells [26]. Th17 cytokines independently induce osteoclast differentiation from their precursor [17,18,27]. In this study, when PB monocytes were cultured in media supplemented with Th17 cytokines and SKI306X, SKI306X reduced Th17 cytokine-induced osteoclast differentiation. The underlying mechanism of action of this drug is unclear; hence, further studies are required to determine the cell receptors and signaling pathways involved herein.

Monocytes express various chemokine receptors including C-C chemokine receptor type 2 (CCR2) and (C-X3-C motif) chemokine receptor 1 (CX3CR1) and interact with chemokine ligands expressed by synovial fibroblasts. Their interaction promotes cellular activation, migration, and recruitment into the synovium of RA patients [26]. To investigate their interactions, we co-cultured monocytes with RA synovial fibroblasts under osteoclast-differentiating conditions. RA synovial fibroblasts can potentially augment osteoclastogenesis [28], and TNF-α- or IL-17-stimulated synovial fibroblasts are more effective at osteoclastogenesis [17]. Osteoblast differentiation was augmented when RA synovial fibroblasts were stimulated with IL-17 and then co-cultured with monocytes rather than non-stimulation with IL-17. However, SKI306X restored IL-17-augmented osteoclast differentiation in stimulated synovial fibroblasts. These results suggest that SKI306X reduces osteoclastogenesis through its effects on both osteoclast precursors and cellular interactions with synovial fibroblasts.

There are only two clinical studies of SKI306X in RA; however, the studies have limitations regarding the assessment of the clinical efficacy of SKI306X in RA patients. One study is a six-week, double blinded noninferiority study for assessment of pain relief and tolerability of SK1306X compared with celecoxib. The duration of the study is too short to assess clinical efficacy and protective effect of joint destruction. Because SKI306X is compared with celecoxib rather than DMARDs, the disease modifying effect of SKI306X for RA cannot be assessed [10]. The other study evaluated cardiovascular risk associated with SKI306X use in RA patients which was compared with celecoxib and naproxen. A total of 27,253 patients were studied and the incidence of major cardiovascular events was highest for celecoxib (15.4%), followed by SKI306X (8.6%) and naproxen (8%). SKI306X did not have a higher risk of cardiovascular events than naproxen. This study is a retrospective observational study using data from National Health Insurance Service–National Sample Cohort and it does not assess the therapeutic efficacy of SKI306X. However, this study has a meaningful result because the cardiovascular risk is high in RA patients and it influences their survival [11]. RA is associated with high cardiovascular risk, affecting patient survival, and inflammation and atherosclerosis are closely linked; the pathological features are similar in both disease states and they share common risk factors [29]. Furthermore, the overall risk of metabolic syndrome is higher in patients with RA than in healthy controls and RA is associated with body weight changes, dyslipidemia, adipokine profile changes and insulin resistance in metabolic syndrome [30]. Although IL-17 has a double-sided effect in atherosclerosis, IL-17 could be involved in the process of atherosclerosis of RA. It induces the release of chemokines and their ligands (chemokine (C-X-C motif) ligand (CXCL)1, CXCL2, CXCL8 and CXCL10), which recruit neutrophils and monocytes to the atherosclerotic lesion. IL-17 simulates monocytes to produce IL-6, TNF-α and IL-1β, which enhance plaque instability [31,32]. In early atherosclerosis, increased carotid intima-media thickness is associated with the IL-17-related chemokine eotaxin [33], and in RA patients, IL-17 is the main predictor of microvascular function and arterial compliance, suggesting a significant role for IL-17 in increased cardiovascular risk in RA [34].

This study is the first to report that SKI306X regulates RANKL production and osteoclast differentiation in RA. The mechanism of action of SKI306X is unclear, unlike that of conventional DMARDs. Recently, targeted or biologic DMARDs have been preferred in treating active RA; however, their usage in combination with SKI306X potentially results in in additional therapeutic effects. There have been no clinical or experimental studies of the comparison between SKI306X and other DMARDs such as methotrexate or hydroxychloroquine. There are only comparative clinical studies of SKI306X for assessment of pain relief, tolerability and cardiovascular risk. Based upon the results of this study, a comparison study of the therapeutic effects of SKI306X versus DMARDs and the assessment of combination effects of SKI306X with DMARDs in RA patients should be performed in the future.

## 5. Conclusions

SKI306X reduced both inflammation and osteoclast differentiation in RA, reducing Th17 cytokine-induced TNFα and IL-1β expression and production in RA synovial fibroblasts during inflammation. SKI306X ameliorated RANKL production in synovial fibroblasts and osteoclast differentiation in circulating monocytes. SKI306X is thus a potential novel therapeutic agent to prevent inflammation and joint destruction in RA.

## Abbreviations

Th17	T helper 17
IL-17	interleukin 17
IL-21	interleukin 21
IL-22	interleukin 22
RA	rheumatoid arthritis
FLSs	fibroblast-like synoviocytes
TRAP	tartrate-resistant acid phosphatase
TNF-α	tumor necrosis factor-α
IL-1β	interleukin-1β
M-CSF	macrophage colony-stimulating factor
RANKL	receptor activator of nuclear factor kappa-B ligand
